# Mass Spectrometry Coupled Experiments and Protein Structure Modeling Methods

**DOI:** 10.3390/ijms141020635

**Published:** 2013-10-15

**Authors:** Jaewoo Pi, Lee Sael

**Affiliations:** 1 Department of Computer Science, Stony Brook University, Stony Brook, NY 11794, USA; 2 Department of Computer Science, State University of New York Korea, Incheon 406-840, Korea; E-Mail: jwpi@sunykorea.ac.kr

**Keywords:** constraint-base structure prediction, integrative structure prediction, sequence variants, protein dynamics, mass spectrometry

## Abstract

With the accumulation of next generation sequencing data, there is increasing interest in the study of intra-species difference in molecular biology, especially in relation to disease analysis. Furthermore, the dynamics of the protein is being identified as a critical factor in its function. Although accuracy of protein structure prediction methods is high, provided there are structural templates, most methods are still insensitive to amino-acid differences at critical points that may change the overall structure. Also, predicted structures are inherently static and do not provide information about structural change over time. It is challenging to address the sensitivity and the dynamics by computational structure predictions alone. However, with the fast development of diverse mass spectrometry coupled experiments, low-resolution but fast and sensitive structural information can be obtained. This information can then be integrated into the structure prediction process to further improve the sensitivity and address the dynamics of the protein structures. For this purpose, this article focuses on reviewing two aspects: the types of mass spectrometry coupled experiments and structural data that are obtainable through those experiments; and the structure prediction methods that can utilize these data as constraints. Also, short review of current efforts in integrating experimental data in the structural modeling is provided.

## 1. Introduction

**Figure 1:**
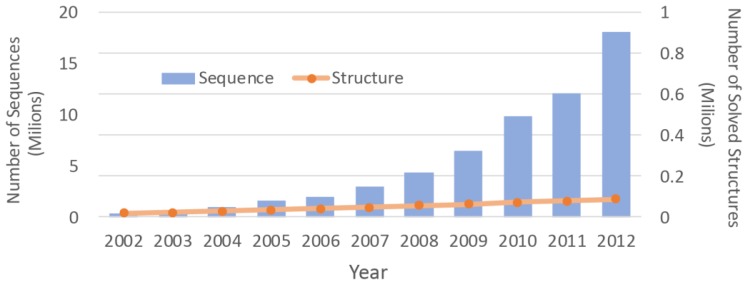
Number of solved structures *versus* number of identified protein sequences. Numbers of sequences and protein structures are obtained through Uniprot (http://www.ebi.ac.uk/uniprot/) and RCBS PDB (http://www.rcsb.org), respectively.

In the post-genomics period, more researches are focused on functional and conformational analysis of proteins in a genomic scale [1]. Although experimental methods, such as nuclear magnetic resonance (NMR) spectroscopy and X-ray crystallography, have advanced in the past two decades, these methods are still labor intensive, high cost, and it can take weeks to months to solve a three dimensional structure [2]. Due to the difficulties associated with the experimental methods, the number of protein structures that have been solved is much smaller than the number of protein sequences. With advancements in the sequencing machines, the gap between the numbers is growing even faster (Figure 1). Structure prediction approaches can be used to overcome this chasm. By determining three dimensional (3D) molecular structures [3,4], they can be used to analyze structural interactions between biomolecules [5,6] and to determine the functionality of a protein or protein complexes [7,8]. However, prediction of precise structures in the presence of variations (or mutations) remains challenging. Determination of their atomic level dynamics also remains difficult.

Integration of proteomics results, such as mass spectrometry (MS) coupled experiments, can reduce the difficulties associated with structural modeling. MS-coupled methods such as hydrogen-deuterium exchange (HDX), hydroxyl-radical mediated covalent labeling (protein footprinting), chemical cross-linking, ion mobility spectrometry, and native methods have emerged as structural proteomics techniques for analyzing the protein complexes, for identifying structural change up-on binding, and for detection of post-translational modifications. MS-coupled experiments provide fast and highly sensitive spatial information of the structure being analyzed. Much of the spatial information can be integrated into the structure prediction methods. They can be used to choose the structure that is most consistent with the MS-coupled experiments. They can be also used directly in the structure optimization procedure. MS-coupled methods, in addition to being fast and highly sensitive, require less mass of sample to extract the structural information compared to traditional structure solvers. This means that multiple experiments can be done without being limited by the available sample.

Although there are many studies on both the MS-coupled experiments and the structure prediction methods, the integration of the experimental data with the computation methods is still not widely explored. Developments in the integrative methods will provide advancements in the structural biology area. For this reasons, this review focuses on the mass spectrometry for studying the structural and dynamics of biomolecules [9,10] and structure prediction methods to promote integrative method development and researches in structural bioinformatics. The review is organized as follows. First, types and characteristics of MS-coupled experiments are overviewed. Then, a review of structure prediction methods is provided. In the last section, existing integrative methods are described with suggestions for further integrations.

## 2. Mass Spectrometry Techniques

Mass spectrometry experiment (MS) is a high-throughput experimental method that characterizes molecules by their mass-to-charge (*m*/*z*) ratio. The MS is composed of sample preparation, molecular ionization, detection, and instrumentation analysis processes [11]. MS is beneficial in that it is generally fast, requires a small amount of sample, and provides high accuracy measurements. For these reasons, MS alone or combined with other structural proteomics techniques is widely used for various molecular biology analysis purposes. Examples of the analysis include post-translations modifications in proteins, identification of vibrational components in proteins, and analysis of protein conformation and dynamics [12]. We will focus on MS-coupled methods that provide information about conformation and dynamics of the protein being studied (Table 1). For a comprehensive review on MS procedures, refer to [12], and for a review on various types of MS-coupled methods, refer to [9].

### 2.1. Hydrogen/Deuterium Exchange Mass Spectrometry

Hydrogen/deuterium exchange mass spectrometry (HDX-MS) exploits the chemical exchange pattern of amide hydrogens, *i.e.*, hydrogens that are attached to the backbone nitrogen in proteins [13]. In a HDX experiment, proteins are placed in a solution containing deuterated water (D_2_O). Inside the solution, the amide hydrogens (H) exchange with the deuterium (D). This exchange increases the mass of proteins. The proteins can then be treated for the MS analysis to find out the overall mass change. Alternatively, the protein can be fragmented and fragments can be treated for the MS analysis to find out the mass change for each of the fragments.

The location and rate of the exchange depends on the solvent accessibility, hydrogen-bonding, pH level, and temperature. Assuming that the pH level and the temperature can be controlled, the solvent accessibility and hydrogen-bonding can be detected through analysis of the change in mass. The hydrogen exchange event occurs primarily in the amide hydrogen of residues on the solvent accessible region of the protein. However, not all solvent accessible residues have amide hydrogen available for the exchange event. Amide hydrogen also plays a role in constructing secondary structures such as alpha-helices and beta-sheets. When a secondary structure is formed, hydrogen bonding occurs between amide hydrogen and electro-negative atom in the side chains of other residues. A stable structure makes hydrogen exchange in the amide hydrogen less likely.

Depending on the availability and stability of the (local) structures, the rate of the exchange differs. Amide hydrogens that are exposed on the surface exchange hydrogen with deuterium quickly, while those buried in the core have much slower exchange rates. For the amide hydrogens that are solvent accessible but are part of hydrogen bonding, the exchange happens much slower through low-frequency vibration motions of the proteins.

Some of the successful applications of HDX include detecting binding affinity between HIV-1 Nef and Lyn SH3 [17], detecting conformational dynamics of the scaffold protein in the presence and absence of lipid [18], and examining the structural changes in the binding cites of the vitamin D receptor when bound to its natural ligand, 1α,25-dihydroxyvitamin D3, and two analogs ligands, alfacalcidol and ED-71 [19].

### 2.2. Hydroxyl-Radical Mediated Covalent Labeling Mass Spectrometry

Hydroxyl-radical mediated covalent labeling, or protein footprinting, is a MS-coupled technique that is conceptually similar to HDX-MS. Similar to HDX-MS, protein footprinting also probes the solvent accessible residue and makes modifications to the accessible residues. The major difference between the HDX-MS and the protein footprinting is that HDX-MS targets the backbone amide hydrogen whereas the protein footprinting targets the side chains of the residues. In the protein footprinting, relative hydroxyl radicals, which have water-like solvent properties, interact with the side-chains of the solvent accessible residues and form stable covalent modifications that are detectable by MS [14]. More specifically, side-chains of the solvent accessible residues are exposed to hydroxyl radicals and undergo covalent oxidation. The oxidation of the side chains results in mass shift which can be detected by MS. The comparison between the unmodified and modified proteins reveals which residues are solvent accessible [10]. Protein footprinting provides a more direct measurement of solvent accessibility compared to the HDX-MS experiments.

The location and rate of the oxidation differs depending on the solvent accessibility of the residues and the reactivity of the side chains to hydroxyl radicals. Solvent accessibility of the protein structures can be evaluated through analyzing the correlation between the accessibility and the oxidation level for each type of amino acid [20]. The relative reactivity of residue to hydroxyl radical depends on the side-chain chemistry, that can be listed by order of reactivity as follows: Cys > Met > Trp > Tyr > Phe > Cystine (two disulfide bonded Cys) > His > Leu ~ Ile > Arg ~ Lys ~ Val > Ser ~ Thr ~ Pro > Gln ~ Glu > Asp ~Asn > Ala > Gly [21]. Of these residues, Gly, Ala, Asp, and Asn have low reactivity, and thus, are not useful. In addition to the reactivity, mass change after oxidation needs to be large enough for MS to detect. For this reason, although Ser and Thr are reactive, they cannot be used for detection of solvent accessibility. In summary, 14 residues out of the 20 amino-acids can be used to detect structural properties via the protein footprinting method [22].

Detection of solvent accessibility enables the protein footprinting to be an attractive method for identifying interaction regions [23]. One of the first uses of protein footprinting was in characterizing DNA-protein interactions such as detecting sequence-specific interactions of I12-X86 lac repressor with non-operator DNA [24]. Protein footprinting has also been used to study the structural aspects of transmembrane proteins such as G protein-coupled receptors. In one study, protein footprinting was used to provide evidence that water molecules embedded and conserved in the G protein-coupled receptors are likely to be functionally important [25].

However, the preferential interaction quality makes the analysis of the MS results challenging. In order to apply the protein footprinting method for solvent accessibility analysis, accurate analysis of the correlation between the solvent accessibility and the reactivity of the residues is needed [10]. For further details on various protein footprinting techniques, readers can refer to a review by Kiselar and Chance [14].

### 2.3. Chemical Cross-Linking

Chemical cross-linking combined with a MS analysis is another important proteomics technique for structural analysis. Chemical cross-linking experiments are used to detect spatial closeness between residues in a protein for structure analysis purposes. They are also used to detect interacting region between proteins [15]. Chemical cross-linking involves the use of a special reagent called cross-linkers, most often lysine linkers, to covalently attach two residues within a protein or between proteins that are spatially close. After the chemical cross-linkage process, MS analysis is performed to detect the cross-linked regions [14]. The identified cross-link location information can be transferred to as distance constraints between residues. A sufficient number of distance constraints is known to provide important clues about the 3D structure of the protein.

**Figure 2:**
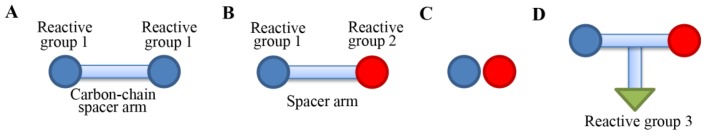
Four types of cross-links (adapted from figure 3 of [26]). (**A**) Homo-bifunctional; (**B**) Hetero-bifunctional; (**C**) zero-length; and (**D**) hetero-trifunctional cross-link.

Cross-links are generally formed by chemical reactions that are initiated by various factors, such as change in pH, heat, and radiation. The type of activator differs by the type of cross-linking reagents and results in cross-links of different characteristics. Figure 2 shows four types of cross-linking reagents in a cartoon form. In a homo-bifunctional cross-linking, two of the same types of reactive groups are linked by a carbon-chain spacer arm (Figure 2A). In a hetero-bifunctional cross-linker, two different types of reactive groups are linked by a spacer arm (Figure 2B). In a zero-length cross-linking, cross-linking agents mediate amide or a phosphoramidate bond formation of the two reactive groups without the intermediate spacer (Figure 2C). The zero-length cross-linker is especially useful when we want to detect residues that are within 3Å in space. There is also a hetero-trifunctional cross-linking agent, where three types of reactive group can be cross-linked. In the trifunctional cross-linking, a third reactive group from a protein can be attached or can be used for affinity purification purposes in case a biotin moiety is incorporated [26].

Cross-linking coupled MS has been successfully used to determine interactions between proteins. It has been used to identify interaction sites between heat shock protein and substrates [27], determine the structural organization of 19S regulatory particles in the 26S proteasome [28], and assess dynamic structures of viral capsid by identifying residue specific inter- and intra-subunit interactions in the viral capsid precursor [29]. There are various advantages of chemical cross-linking experiments including the importance of the distance constraint information obtainable from the experiment and the ease of the cross-link experiment. However, due to the complexity in the cross-linking chemistry, the MS analysis is considered to be challenging and requires advances in both the experimental and computational analysis strategies [14]. A survey of chemical cross-linking technique can be found in a review by Sinz [26].

### 2.4. Ion Mobility-Mass Spectrometry

Ion mobility-mass spectrometry (IM-MS) is a multi-dimensional separation method that combines the ion-mobility spectrometry experiment with the MS experiment to identify components in the test sample. The major contribution of IM-MS in the proteomics studies is the capability to separate molecules by their size and shape, which enables the discrimination and determination of heterogeneity in the biomolecules [16].

In the IM-MS process, the ion mobility spectrometry experiment (IM) separates the initial batch of ionized test sample according to their mobility in the gas phase. The mobility depends on the size and shape of each ion. Other factors, such as structural heterogeneity and flexibility that effects the orientation and distribution of charges on the ion, also play important roles in the mobility of ions [16,30]. However, comprehensive list of factors and their mechanisms are not yet known. Known factors are controlled and utilized to analysis the characteristics of the molecule. After IM process, the ions are further separated by their mass-to-charge ratio (*m*/*z*) by the MS analysis. The MS process, in most case, is done in vacuum conditions and utilizes the distinctive properties of ions to determine their mass.

Since IM-MS experiment is executed in gas and vacuum states, the molecules being studied are more dynamic compared to when they are in a crystalline state, which is a required state for X-ray crystallography. This property allows for better analysis of the dynamics of the proteins being studied as well as providing more native-like information about the fold of the proteins [31]. Also, diffusion cross-section data obtained in the IM process provides information about the radius of gyration of the protein [32].

IM-MS has been successfully used to identify the ring-like topology of trp RNA binding protein, composed of 11 members, by determining its collision cross section[33] to study the relative population of oligomers of 42-residue amyloid beta-protein and its alloform with 19th residue substituted to proline [34], and to characterize the oligomeric population detected during the formation of fibrils of β(2)-microglobulin. This helped to identify the properties of transient, oligomeric intermediates formed during assembly of the fibrils [35]. Diverse types of IM and MS exist that can be combined to form the IM-MS technique. A review on the types of IM methods coupled with MS can be found in [36]. Also, further description and application of IM-MS method in the context of applications to structural biology can be found in [16].

### 2.5. Native Mass Spectrometry

Native MS is a group of MS-coupled experiments that focuses on the structure, dynamics, and subcomponent interaction of intact biomolecular complex in a native-like state [37]. Native MS is often combined with various MS-coupled methods, such as electrospray ionization MS (ESI-MS) and ion mobile MS (IM-MS), and structure optimization programs to determine the topology and dynamics of quaternary structures in their native-like state [38]. Native MS in itself is a low resolution structure determination technology. However, compared to the traditional structure determination technologies such as X-ray crystallography and NMR, it is more sensitive, faster, and allows higher selectivity as well as providing information on stoichiometry, stability, and spatial arrangement of the subunits in the complex [38,39]. The higher sensitivity comes from the environmental property of native MS that preserves the native-like conditions of the native structure and dynamics of the complex.

There is diversity in the methodology and the application of the native MS. However, only the key characteristics are pointed out to enhance understanding of its usefulness in structural modeling. Native MS has special properties such as non-denaturing ionization of electrospray ionization (ESI) [40]. The electrospray ionization of native MS involves the dispersion of the liquid state solution into nano-droplets which are then reduced to maximal surface charge of molecular till a certain size and composition is reached. Then, the ion-free state is accomplished through uses of volatile ESI compatible buffers under native-like conditions. More details of the electrospray ionization process can be found on review by Kebarle and Verkerk [41]. After this process, the complex can be decomposed to sub-complexes and subunits. Denaturing MS can be used to find the mass of subunits and subcomplexes, revealing the topology of the complex [40]. Tandem MS can be used additionally to validate the subunits and also to identify peripheral subunits. Ion mobility MS is a rather young addition to the native MS pipeline that can be used to determine the shape and cross-section of intact complexes and subcomplexes [38,42].

Native MS has been used to characterize the structure of 20S proteasome [43,44], confirm the subcomponents and stoichiometry of RNA polymerase II and III [45], and study the endogenously expressed protein complexes including exosome [46]. More details in the application of native MS for structural analysis will be described in Section 4.

## 3. Structure Prediction Methods

In this section, we focus on the structure prediction methods which often act as prerequisites of function annotation of protein or protein complexes. We take special interest in properties that have the potential for being integrated with the MS experimental data for sensitive modeling of structure and dynamics of biomolecules of interest.

Protein structure prediction falls into three categories depending on the availability of solved structures: homology (comparative) modeling, threading (fold recognition), and free (*ab initio*) modeling [47]. Comparative modeling builds a model using experimentally solved 3D structures (templates) that have high sequence similarity to the protein being analyzed. Threading involves the alignment of the target sequence directly to 3D structures of proteins utilizing structural and biochemical similarities detectable between the target sequence and 3D structures in the database. This allows for relaxation of sequential similarity between the target and the template. Free modeling, or the *ab initio* method, predicts a model without a template structure, utilizing the force fields and knowledge-based potentials of the target sequence. Table 2 summarizes the three types of modeling methods.

### 3.1. Homology Modeling

The structure prediction process of homology modeling, according to Martí-Renom *et al.* [59], is composed of four sequential steps: (1) fold assignment and template selection; (2) target-template alignment; (3) model building; and (4) model evaluation. Templates are selected based on the sequence similarities that are analyzable after the sequence alignments. The first two steps can be executed together using fast but accurate alignment methods. It has been shown that homology modeling can achieve accuracy up to backbone RMSD of 1–2 Å when a template of 50% or higher sequence identity is found and used [60].

Template selection and alignment are two of the most important components in comparative modeling. Thus, development of sequence alignment methods with high sensitivity and specificity is critical [61]. Template selection and alignment methods have evolved towards improving the balance between the two criteria. Earlier approaches used pairwise alignment methods, such as FASTA [62] and BLAST [63], to compare sequence similarity between target sequence and sequences on the database. Nowadays, multiple sequence alignments are being used. Multiple sequence alignments are shown to improve the sensitivity of alignment without sacrificing the selectivity. Multiple sequence alignment also has been shown to be better in preserving structural similarities [64]. They are also used to find highly conserved region, such as ligand binding sites. Some of the available multiple sequence alignment tools are MUSCLE [65], ClustalW [66], PSI-BLAST [67], and HHsearch (as part of the HH-suite [50]).

Once the model has been aligned, the next step is the model building. This involves initial assignment of Cartesian coordinates to the target. The idea of conventional model building started from copying 3D coordinates from a database of templates. The easiest, yet widely used approach is called rigid body assembly. In the rigid body assembly, first a conserved core region from a small number of templates is constructed by superposing and averaging coordinates of Cα atoms or backbone molecules [68]. After initial assignment is made, the model rebuilds non-core regions such as side chains. Loop regions are often optimized further since the structure of those areas are less conserved [69,70]. Alternative model building methods utilize the segment-matching approach. The segment-matching approach is an extension of rigid body assembly that utilizes the coordinates of small segments that best align with the protein of interest. Unger and co-workers [71] introduced and experimented the “building blocks” approach on hexametric structures. The building block approach first builds a model from the representative segments (blocks), then replaces them by another segment within the cluster whose RMSD is smaller. Similarly, Levitt [72] first divided the target sequence into short segments, then matched fragments from the database using energetic or geometrical criteria, which are: Sequence similarity, conformational similarity (secondary structure and atomic coordinates), and compatibility (van der Waals interactions). Modern homology modeling has evolved into much more sophisticated approach, conjoined with global energy minimization procedure. This approach is called modeling by satisfaction of spatial restraints [59,70].

### 3.2. Threading

Threading shares many methodological similarities with that of homology modeling. The difference lies in the properties used for target-template alignment. Unlike homology modeling that relies solely on the sequence information, threading aligns the target protein sequences and target structures by their statistical similarity between sequence and structural properties. This idea expanded from the observation that the diversity of sequences is higher than that of the folds. An earlier threading approach by Bowie *et al.* [73,74] introduced the sequence-structure profile matching method. The method generates structural profiles from the environmental factors of the residues in the 3D structure. The environmental factors include the area of the residue buried in the protein which is inaccessible to solvent, the fraction of side-chain area that is covered by polar atoms, and the local secondary structure. The 3D profiles are aligned with dynamic programing based on the statistical compatibility with the 1D target sequence independent of the template sequence information.

One of the representative threading algorithms, PROSPECT [75], utilizes residue-residue contacts information. PROSPECT finds globally optimal threading alignment between the target sequence and the template structure with a divide and conquer approach. It first divides the template into small substructures in the form of a tree, and then an iterative procedure of alignment and local optimization is performed until the whole template is considered and the total energy is minimized. Their scoring function is a weighted linear function consisting of four energy terms [76]: mutation, singleton, pair-contact potential, and alignment gap penalty. The mutation energy term is a compatibility measurement for substituting the template amino acids by target acids. The singleton energy term measures the compatibility of aligning the target amino acid onto the template position base. More specifically, the singleton term examines the likelihood of substituting one residue to another and the preference of secondary structure and solvent accessibility for the particular residue. Pairwise-contact potential energy is a statistical term reflecting the likelihood of the residue of types *i* and *j* to be in contact, *i.e.*, resides within 9Å but separated by three or more residues in sequence. The alignment gap penalty energy term gives more penalties to larger gaps in alignment.

One exemplar of recently developed threading algorithm is MUSTER [52]. MUSTER also uses dynamic programming to identify the best match between the target and the template sequences. The scoring function of MUSTER consists of seven energy terms. The first term is the sequence profile, which denotes the frequency of the residue types at a position in template and can be acquired by PSI-BLAST multiple sequence alignment. The second term indicates secondary structure match between the residues of the target, predicted by PSI-PRED, and the analyzed secondary structure of the residues of the template. The third term is the structure profile that is derived by a depth-dependent structure analysis. The depth of the residue is the measurement of the depth from the protein surface to the residue by calculating average distance of a residue from the solvent water molecule. Unlike solvent accessibility, it can distinguish atoms just below the surface and those in the core [77,78]. MUSTER splits the initial templates with nine residues by a gapless threading. Then fragments with similar depth (those with smaller RMSD) from the database are collected to calculate the frequency profile at each position of the template. Fourth term is solvent accessibility term, which compares the solvent accessibility of the template assigned by STRIDE [79] and the solvent accessibility of the target predicted by the two-state neural network machine. The fifth and sixth term assigns scores based on the similarity between the two torsion (psi and phi) angles of the template and the torsion angles of the target predicted by support vector regression. The last term is from hydrophobic scoring matrix, matching the hydrophobic patterns of target and template.

### 3.3. *Ab Initio* Method

*Ab initio* method, alternatively called *de novo* or free modeling, is a structural modeling approach that does not rely on template structures. Although homology modeling and threading can achieve higher prediction accuracy, *ab initio* methods are needed when there are no detectable template structures in the database. There have been numerous advances in the *ab initio* methods. However, computation time cost is still high and building models with more than 150 residues are still challenging in terms of accuracy [4,60].

There are two directions in the *ab initio* methods, one is more physics-based and other is more knowledge-based. Physics-based methods are generally more interested in the fold dynamics themselves while knowledge-based methods are focused on the accuracy of the final structure. Physics-based methods are often integrated with molecular dynamics simulations using physics-based force fields. Representative examples of modeling systems using all-atom physics based force fields include CHARMM [80], AMBER [81], and OPLS [82]. Their force fields share potential terms including intra-molecular terms such as bond lengths, angles and torsion angles, as well as non-bonded terms such as Coulomb potential and Lennard-Johns. Knowledge-based methods are focused on the resulting structure rather than the actual fold mechanism. For this reason, they use knowledge-based potential energy functions in addition to simple energy terms. Also, reduced models of the residues are often used to speed up the computation and increase the conformational search space [58,83]. Knowledge-based *ab initio* methods rely on the efficient structure space sampling algorithms as well as the effective scoring functions. It is not feasible to consider all possible conformations a structure can have. Thus, often variants of Monte Carlo sampling methods are used to search for possible conformations. Scoring function integrated with the sampling methods is also important for finding the most native-like structures. Following are some of the energy terms used in the scoring functions of *ab initio* methods, including SimFold [56] and QUARK [4].

#### 3.3.1. Backbone Torsion Angles (Dihedral Angles) Potential

Many structure prediction approaches take advantage of statistically probable phi/psi angle distributions. Ramachandran plot—*i.e.*, plot of psi and phi angle present in a structure—is useful tool for visualizing the torsion angles of a conformation and determining if they fall into a native-like psi-phi distribution. Both SimFold and QUARK defined this energy function as the sum of probability of phi and psi angles:

(1) $$E_{dh}=-\sum_{ResidueLength-2}\text{log }P(\varphi ,\psi )$$

Weights in SimFold depend on the type of amino acids and their position in the “quadrant” bin of a Ramachandran plot. Each quadrant corresponds to alpha-helix, beta-strand, alpha_L_, and rare regions [56]. In QUARK, probabilities of phi and psi angles are conditioned on residue type and secondary structure type. For this purpose, 60 different Ramachandran plots of each condition pairs are generated (20 amino acid types × 3 secondary structure types) and used [4].

#### 3.3.2. Hydrogen Bond Potentials

Hydrogen bonds are one of the dominant energy factors for forming the secondary structure and the global topology of a protein structure. SimFold defines the hydrogen bond potential as the summation of following terms: (i) hydrogen bond interaction between any two atoms in the backbone (N, Cα, C); (ii) four-body hydrogen bond characteristic in the β-sheet, which incorporates two hydrogen bonds in neighboring β-sheets; and (iii) the Born- or Self-energy term which is effected by charged and polar groups that determines the propensity for residue to be buried or exposed to solvent [84]. In contrast, QUARK algorithm does not compute hydrogen bonds directly. Instead, QUARK utilizes the geometric features governed by the hydrogen bones between the two closest by residues *i* and *j*: the distance between O*_i_* and H*_j_*, the inner angle between C*_i_*, O*_i_*, and H*_j_*, the inner angle between O*_i_*, H*_j_*, and N*_j_*, and the torsion angle between C*_i_*, O*_i_*, H*_j_*, and N*_j_* [4].

#### 3.3.3. Solvent Accessibility

Solvent accessibilities are the extent to which a protein structure interacts with the solvent [85]. Explicit computation of the solvent accessibility involves various types of factors including electrostatic potential, hydrophobicity, and van der Waals force. Thus, the calculations are computationally intractable and require approximation algorithms. Provided the protein structures, solvent accessibility of biomolecules is often inferred by calculating the solvent-accessible surface area (SASA) or per-residues solvent-accessibility surface area (rSASA) of the structure. The typical method of calculating the precise SASA is done by rolling spherical probe around the bimolecular. The probe size is often 1.4 Å to represent the size of water molecule. rSASAs are often divided by the surface area of each type of residue after assigning the SASA for each of the residues of the biomolecules. Readers interested in extensive discussion of the SASA calculation methods are referred to work by Durham *et al.* [85]. QUARK estimates the solvent accessibility in their optimization process while SimFold does not explicitly account for them in their scoring function [4].

There are also several other energy terms used such as van der Waals interaction, solvation, radius of gyration, and secondary structure packing. Also, spatial constraints are used to avoid collisions, to preserve distance between residues, and to form globular structure. In an integrative structural modeling, energy and spatial constraints can be obtained through experiments instead of prediction from sequences. Application of experiment data will thus increase the accuracy of modeling.

### 3.4. Composite Protein Structure Prediction

Recently, many structure prediction methods consist of a combination of all three types of structure prediction methods [60]. In homology modeling and threading, modification of unconfident regions such as loops are done in *ab initio* fashion. Also, many *ab initio* approaches have adapted the uses of spatial restrains or structural fragments detectable by threading [4,86]. Threading relies more on multiple sequence alignment and sequentially conserved properties to align the sequence to structures. In general, a composite protein structure prediction will first search the template library to determine the availability of homolog structures. If the templates are found, coordinates are assigned to aligned regions between the target and template. Unaligned regions and evolutionarily diverse regions are modeled by *ab initio* methods. If the templates are not found, *ab initio* modeling is performed on all the areas. After the initial prediction, models are evaluated and selected. Then, the full atomic coordinates of side-chains are assigned and optimized [60].

**Figure 3:**
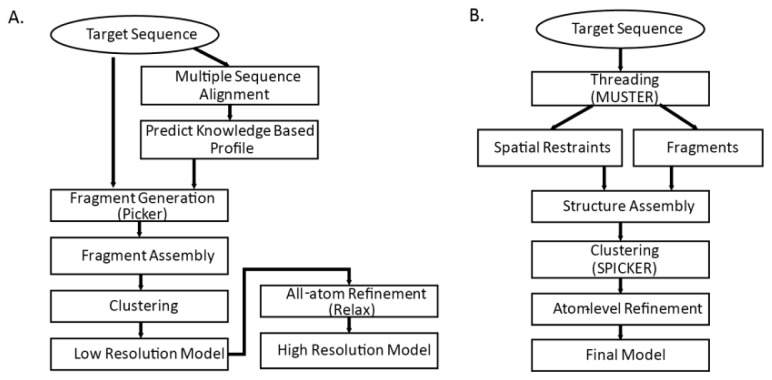
Structure prediction pipeline (**A**) Rosetta [54]; and (**B**) I-TASSER pipeline (adapted from Figure 1 of [55]).

We take a closer look at two structure prediction pipelines of the top CASP predictors: I-TASSER [4,87] and Rosetta [54,86]. Both methods are threading-integrated model free structure prediction methods. The flowcharts of the two methods are shown in Figure 3. Common steps for both methods are fragment generation process, modeling assembly, and atom-level refinement.

#### 3.4.1. Fragment Generation

Sophisticated threading algorithms are used to generate and score target-template alignments. Many algorithms use a combination of both sequence and structure information. Fragments are generated for each segment of the query sequence using the profile that best aligns the sequence segments [4]. I-TASSER builds fragments with continuous lengths from 1 to 20; Rosetta builds possible 3-residue and 9-residue fragments for each of the sequence segments. In Table 3, we show terms used in the scoring function of Picker [88] from Baker’s group and MUSTER [52] from Zhang’s group.

#### 3.4.2. Initial Model Assembly

Reduced model of protein is generally used in the initial assembly. With knowledgebase force field and efficient search algorithm, conformational search is done by Monte Carlo algorithms that iteratively update and optimize confirmation to native structure by energy function. I-TASSER model assembly starts from single decoy and generates many reasonable (*i.e.*, global energy is low and close to zero) decoys by fine tuning Cα atom positions and torsion angles. In contrast, Rosetta fragment assembly finds combinations out of candidate fragments that minimize global energy. Commonly used energy functions are shown on Table 4. A number of possible models are generated as result of the initial model assembly. Those models are then clustered into few categories and structures in the cluster centroids are chosen for further refinement.

#### 3.4.3. Atom-level Refinement

Detailed backbones and side chains of protein are represented and refined. In the previous step, knowledge based force field that is based on the statistics of the known structures are used. In the atom-level refinement step, realistic potential energy terms are used for model refinement. Other terms such as bond length, angle constraints, steric overlaps and hydrogen-bonding network are also used for refinement.

## 4. Integration of Proteomics Data and Structural Modeling

Computational methods that integrate structure prediction and experimental methods are emerging strategies in the structural biology field. There are notably many efforts in integrating low resolution structure analysis methods with computational methods, such as docking substructure to cryo-electron microscopy (cryo-EM) images and small angle X-ray scattering (SAXS) profiles in structure determination. However, there are not many attempts to integrate MS-coupled experiment with structure prediction. For readers interested in the integrative structure modeling methods using cryo-EM images or SAXS profiles, detailed reviews can be found in [89] and [90], respectively. In this section, we first cover some of the existing researches on the application of MS experiments to structural modeling. Then, we provide suggestions on possible MS experimental results that can be used in the structural modeling process to analyze the structure and dynamics of biomolecules.

### 4.1. Chemical Cross-Linking Experiment Integrated Structure Modeling

Chemical cross-linking based MS experiments that provide information about molecules close in distance are one of the earliest and most intuitive MS-coupled experiments that can be integrated to the structure modeling. Using the result for the chemical cross-linking to extract distance constraints, structure modeling can use the distance constraints to either refine the structures in comparative modeling or use in order to limit the sample space in *ab initio* modeling.

In the early work by Young *et al.* [91], intra-molecular cross-linking, MS and threading are used to identify the structure or the fold of a bovine basic fibroblast growth factor (FGF)-2. Using a lysine-specific cross-linking agent, they identify the eight lysine-lysine links in the FGF-2 that are validated with the MS. With the distance constraints from the cross-linking experiment combined with the threading method, they were able to correctly identify the fold type of FGF-2 as the b-trefoil fold. They were also able to model the FGF-2 with homology modeling with backbone RMSD of 4.8 Å.

Chemical cross-linking has been applied for determining the topology of macromolecules that are difficult to detect by the traditional structure solution techniques. Chen *et al*. [92] applied the chemical cross-linkage information to determine the architecture of RNA polymerase II with the transcriptional initiation factor (TFIIF) at a peptide resolution. With the cross-linking coupled with the MS, they were able to identify 253 inter-protein and 149 intra-protein links. The subcomponents of the complex were predicted by homology modeling, when the crystal structures are not available. Then, the linkage information was applied to determine the distance constraints used to manually reconstruct the complex using the structures of the 15 subunits.

Chemical cross-linkage can also be used to determine the fold of a single protein. In the work by Fioramonte *et al.* [15], the utility of the intra-protein chemical cross-linking in determining the secondary structure of polypeptides without any homology information was illustrated. They exploit the geometric characteristics of alpha-helix and beta-sheets, such as the tendency of residues with bulky side chains to form beta-sheets and distance between the linkages formations used to derive cross-linking rules. Cross-linkage rules are then used to determine the secondary structure of polypeptides or proteins. More recent researches exploit the technical advances in the cross-linking. Instead of being restricted to lysine cross-linking, cross-linking is now possible between divers residue types. This increases the detectable number of distance constraints. A review on chemical cross-linking applied to structure modeling can be found in [93,94].

### 4.2. Native Mass Spectrometry Integrated Structural Solvers

Native MS is an emerging technique for macromolecular structure determination. As described in the previous section, native MS is a combinatorial method that involves several MS-coupled methods to detect large molecular complexes that are often not detectable by traditional structure determination methods. It is often combined with computational modeling methods to integrate the exiting knowledge of structure with the experimental results. Heck [37] points out that a native MS can be used to bridge the gap between the interactomics—the study of biomolecular interaction—and the structural biology. The study of the interaction of molecules is traditionally performed by yeast two-hybrid screening or by affinity purification MS. These methods are often high throughput and can be applied in massive scales. Although native MS is currently unable to scale up to high throughput studies, both in time and size, it has been often shown to be successful in determining interaction between subcomponents of complex structures. Successful applications of macromolecules, such as virus [95], yeast exosome [46,96], proteasome structure [43,44], RNA polymerase structure [97], and therapeutic antibodies [98], have been shown.

Taverner *et al.* [42] propose an integrative modeling method for identifying the subunit architecture for intact protein complexes using MS and homology modeling. In their method, complex of interest is first isolated using affinity tag and column chromatography. Then, gel electrophoresis and tryptic digestion is performed to determine the subunit composition of the complex. The masses of the complex and identified subunits were then determined by a spectrum of the denatured proteasome lid. Mass of subunits and their stoichiometry was searched against the known units in the database using their search engine, SUMMIT, to identify the actual subunits. Also, interaction network was built using their subunit interaction information. Homology modeling method was used to model the structure of subunits and the structure of the complex was derived manually based on the interaction information obtained through native MS experiment. Then, the structural fit to the experimental result was evaluated.

There are various applications of native MS in analysis of oligomeric structures. Most of the focus is not on computational structural modeling, although homology modeling of subunits is used in several cases [42,99,100]. There are several reviews on native MS and applications to structure modeling [37,38,40].

### 4.3. Multiple-Experiment Combination for Structural Modeling

Lasker *et al.* [101] suggested an automatic iterative four-step integrative structure modeling procedure that can be used to combine experimental methods in structural modeling. The four steps consist of (1) finding available information about the structure of interest; (2) designing systems that will extract spatial restraints from the available experiments; (3) computing candidate structures that satisfy the spatial restraints; and (4) evaluating the candidate structures. They proved the usefulness of this procedure by predicting the architecture of the human RNA polymerase II and verifying the prediction against a known experimentally solved complex. The initial data used, in addition to the experimental data, were 12 homology modeled subcomponents found in the MODBASE [102], proteomics data including affinity capture MS proteomics data for yeast RNA polymerase II subunits extracted from the BioGRID [103], and an electron density map of human RNA polymerase at 20 Å resolution found in the EMDataBank [104], which were processed to extract spatial restraints.

Zhou and Robinson [105] also review how superimposition of high resolution subunits into low resolution complex extracted from various MS experiments, including ion mobility (IM)-MS, and cyro-EM image, can be done. Benesch *et al.* [106] provide a comprehensive review on gas phase (native state) proteomics methods that can be applied to analyze protein complexes.

### 4.4. Constraints Common in MS-Coupled Experiments and Structure Modeling Methods

Structural proteomics is becoming more practical with the advancement of computational models and proteomic methods [107]. However, they are still either experiment-dominant, not exploring the benefits of computation methods, or computation-dominant, being limited by the available experimental data. Also, most experiments are used to find the topology of the complex structure or their structural change altered by binding. However, we argue that experimental methods can also be used to model individual structure focusing on their change in structure and dynamics upon mutation and/or modification. To promote balanced integration of both experimental and computational methods, we identify some of the constraints that can be used to model structures as shown in Table 5.

## 5. Conclusions

Although there have been various efforts for integrating proteomics data into the structural modeling, they are not enough. In this review, we identified and reviewed both the MS-coupled experiments and structure prediction methods such that researchers working on one field (MS or structure prediction) will have a better understanding of the other. Examples of efforts in integrative structure modeling were provided to argue that integrative methods can be successful. However, there are not many methods that are available to directly apply the experimental results in the structural optimization process. There are several reasons for the limitations. One reason is that translating the experimental result to spatial constraints that are addressable by structural modeling has not been investigated enough. Another reason is that availability of the MS-coupled data is limited. More efforts in sharing the MS-coupled data will promote advances in the constraint modeling and also in the integrative structural optimization methods.

To promote the idea of integrating the two methods, we have also listed out some of the constraints that are used in the structure prediction methods and ones that are available through the experiments. By listing out information for both the MS-coupled experiments and the structure prediction methods, we showed that there are still wide possibilities in the marriage between the proteomics studies and the structure prediction. Advances in the constraint modeling methods of experimental data and developments of integrative structural modeling methods that are flexible in integrating various constraints will greatly promote the structural genomics. This in turn will enhance our understanding of biology as well as disease mechanisms that are unable to be detected by genomics alone.

## Figures and Tables

**Table 1:** Types and characteristics of mass spectrometry-coupled experiments.

MS-coupled methods	Types of information detected	Characteristics
HDX [13]	-Solvent accessibility -Binding stoichiometry, -Affinity for protein-ligand interactions	-Exchange target backbone nitrogen
Protein footprinting [14]	-Solvent accessibility	-Labeling reagents target side-chains
Chemical cross-linking [15]	-Distance between protein subunits -Subcomplex topology	-Type of activator differs by the type of cross-linking reagents
Ion mobility (IM)-MS [16]	-Protein complex shape and size -Subcomplex topology -Radius of Gyration	-Analyzed in the gas phase
All four methods	-Conformational change	-Can detect changes on a wide timescale -Requires very little sample -Crystallization is not required

**Table 2:** Structure prediction methods and their limitations.

	Accuracy range	Protein size limit	Structure prediction methods
Homology modeling	1–2 Å	NA	MODELLER [48], SWISS-MODEL [49]
Threading	2–6 Å	NA	HHpred [50], RaptorX [51], MUSTER [52], Sparks-X [53]
*Ab initio*	4–8 Å	150	Rosetta [54], I-TASSER [55], SimFold [56,57], QUARK [4], CABS [58]

**Table 3:** Scoring criteria of two fragment generators.

	Rosetta (Picker)	I-TASSER (MUSTER)
Amino Acid Sequence	●	
Query Sequence Profile	●	●
Secondary Structure	●	●
Chemical Shifts	●	●
Distance Restraints	●	
Dihedral Restraints	●	●
Solvent Accessibility		●

**Table 4:** Energy functions used in structure prediction.

Type	Energy Function	Description
Physics	Van der Waals	Non-bonded Energy
	Electrostatics	Coulomb Potential
	Atomic Bond Length	Equilibrium of Bonds

Knowledge	Backbone Torsion Angle	From Ramachandran Plot
	Hydrogen Bonds	Secondary Structure
	Radius of Gyration	Structure Compactness
	Fragment Distance	Distance between Fragments
	Solvent Accessibility	Tertiary Structure

**Table 5:** Constraints and energy terms and their availability.

Constraints and energy	MS-coupled experiments	Structure prediction methods
Solvent accessibility	HDX, protein footprinting	I-TASSER, QUARK, SimFold, Rosetta, PROSPECT, RaptorX, MUSTER
Pair-wise distance constraints	Chemical cross-linking	I-TASSER, QUARK, SimFold, Rosetta, PROSPECT, CABS
Secondary structure	HDX, chemical cross-linking	I-TASSER, QUARK, SimFold, Rosetta, PROSPECT, RaptorX, MUSTER Sparks-X, Swiss-Model
Radius of gyration	Ion mobility	I-TASSER, QUARK, SimFold, Rosetta
Topology	Ion mobility, chemical cross-linking	I-TASSER, QUARK, Rosetta
